# Chessboard stroke attributed to intracranial atherosclerosis

**DOI:** 10.1055/s-0045-1809406

**Published:** 2025-06-21

**Authors:** Raul Medina-Rioja, Juan Carlos Lopez-Hernandez, Enrique Piña-Rosales, Brenda Dzul-García, Andres Mercado-Pompa

**Affiliations:** 1 Instituto Nacional de Neurología y Neurocirugía “Manuel Velasco Suarez”, Departamento de Urgencias Neurológicas, Ciudad de México, Mexico.; 2 Instituto Nacional de Neurología y Neurocirugía “Manuel Velasco Suarez”, Departamento de Neurología, Ciudad de México, Mexico.; 3 Universidad Nacional Autónoma de México, Facultad de Medicina, Departamento de Integración de Ciencias Médicas, Ciudad de México, Mexico.; 4 Instituto Nacional de Neurología y Neurocirugía “Manuel Velasco Suarez”, Clínica de Enfermedad Vascular Cerebral, Ciudad de México, Mexico.

**Figure 1:**
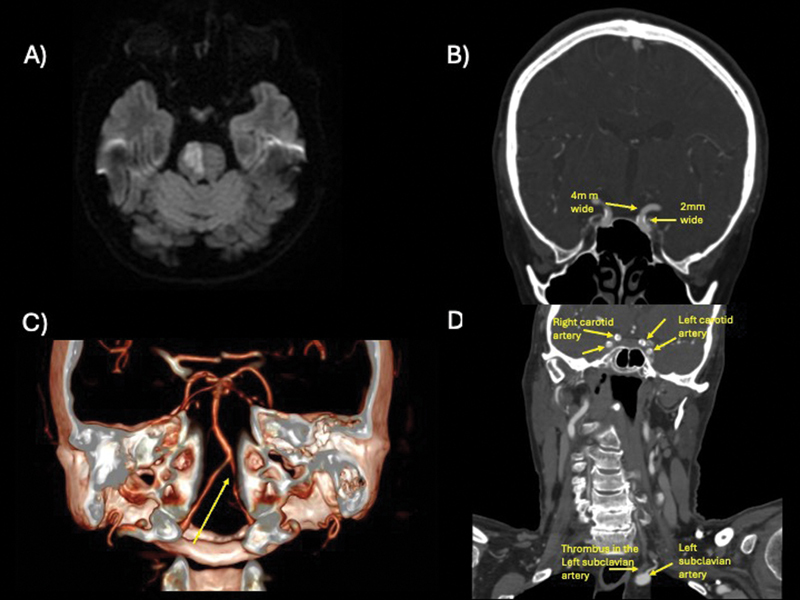
( **A** ) Axial brain magnetic resonance imaging (MRI) scan in diffusion-weighted imaging (DWI) sequence showing an acute stroke located at the right pons. ( **B** ) Coronal angiotomography showing intracranial atherosclerosis in the internal carotid artery, causing 50% of narrowing of the lumen (4 mm versus 2 mm). ( **C** ) Tridimensional reconstruction of neck angiotomography showing left vertebral stenosis at the foraminal segment. ( **D** ) Coronal angiotomography highlighting extra- and intracranial atherosclerosis with a thrombus present in the left subclavian artery.

**Figure 2:**
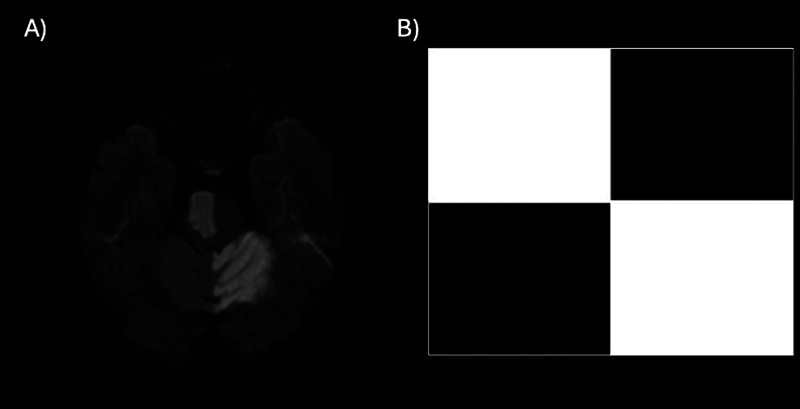
( **A** ) Axial brain MRI in DWI sequence showing two strokes located at the right pons and ( **B** ) the left cerebellum mimicking a chessboard.

A 73-year-old patient presented with sudden double vision and left-sided hemiparesis. A physical exam revealed palsy of the right VI cranial nerve and left arm/leg paresis. A magnetic resonance imaging (MRI) scan showed an acute stroke in the right pons ( Figure 1A ), with the initial workup suggesting intracranial atherosclerosis ( Figure 1B–D ). 1 Other causes were ruled out. The patient was treated with aspirin and clopidogrel and then discharged. He returned the day after with dysarthria and gait instability. A follow-up MRI scan revealed a left cerebellar stroke, creating a chessboard pattern ( Figure 2 ).
